# Role of a regulatory and governance framework in human biological materials and data sharing in National Biobanks: Case studies from Biobank Integrating Platform, Taiwan and the National Biorepository, Uganda

**DOI:** 10.12688/wellcomeopenres.15442.2

**Published:** 2020-09-01

**Authors:** Hellen Nansumba, Isaac Ssewanyana, Micheal Tai, Douglas Wassenaar

**Affiliations:** 1 Central Public Health Laboratories (CPHL), Ministry of Health of Uganda, Kampala, P.O.BOX 7272, Uganda; 2 Chung Shan Medical University, Taichung, Taiwan; 3 Ethics Governance Council (EGC), Taiwan Biobank, Taiwan, Taiwan; 4 South African Research Ethics Training Initiative (SARETI), University of KwaZulu-Natal, Pietermaritzburg, South Africa

**Keywords:** Governance, Biobanking, Data sharing, Biobanks, Biological materials, LMICs

## Abstract

**Background:** In the last decade, Low- and Middle-Income Countries (LMICs) have set up Biobanks to collect human biological materials and associated data for genomic research and public health purposes. Biobanking gives rise to ethical challenges, such as informed consent, benefit sharing, confidentiality, ownership, commercialization and public participation which are harder to navigate in LMIC settings due to disparities in research infrastructure and capacity.  This paper summarizes presentations on Biobank related case studies from two countries, with a focus on challenges in the regulatory and governance framework and suggestions on how to mitigate them.

**Methods: **Two case studies of Biobanks from LMICs have been used **. **The case studies were presented at the 2018 Global Forum on Bioethics in Research (GFBR) meeting on the “Ethics of data sharing and Biobanking in health research”.

**Results: **The case studies show that an integrated, well-regulated platform for human biological materials and data ensures good quality of human biological materials, saves resources and promotes mutual collaboration of work among researchers. National regulatory bodies are required to generate Biobanking guidelines and policies to facilitate guidance to the rapidly changing landscape of science.

**Discussion: **In general, LMICs have weaker research regulatory infrastructure and governance mechanisms for Biobanks than high-income countries. This has increased the fear of exploitation i.e. unfair distribution of risks and benefits. Establishment of Biobanks and producing effective scientific outcomes based on the Biobanking resources is difficult without a proper legislative, regulatory and governance framework.

**Conclusion: **These two case studies from different LMICs settings show that although in both settings there is strong awareness of the scientific and population health value of Biobanks and strong commitment to their establishment, regulatory and ethical guidance show gaps that need to be addressed.

## Disclaimer

The views expressed in this article are those of the author(s). Publication in Wellcome Open Research does not imply endorsement by Wellcome.

## Background

Biobanks have been set up in Low- and Middle-Income Countries (LMICs) in support of research studies on genetic diversity in health and disease in LMIC populations ^1^. The Biobanks have been established to address various research gaps such as National Biobanks, disease specific Biobanks, Biobanking networks etc. ^2– 4^. However, establishment and use of biological materials and data in a Biobank has ethical and legal requirements. The Declaration of Helsinki highlights the ethical principles and governance of Biobanks. It is important to protect the dignity, autonomy and confidentiality of research participants ^5^. Ethical, legal and social issues such as informed consent, benefit sharing, ownership, public engagement and commercialization associated with Biobanks are still complex issues in LMICs. Compared with many high-income countries, where the ethical, legal and social issues of Biobanks have been debated, researchers in LIMCs are less experienced in addressing these issues ^3, 6^.

Data sharing is a key component in Biobanking as they are increasingly being used to support global health research ^7^. Greater data sharing maximizes the value and utility of datasets and minimizes the costs of unnecessary duplication of research ^8^. A study by de Vries *et al*. (2017) on content analysis of ethics guidelines, policies and procedures of 22 African countries indicates that African regulation is either absent, outdated, conservative or difficult to navigate ^9^. Additionally, Research Ethics Committees (RECs) lack guidance on how to review genomics and Biobanking proposals. This paper presents case studies from two LMICs on the regulatory and governance framework for Biobanking, including challenges and suggestions on how to mitigate them.

## Case study 1: Taiwanese experience in Biobanking

### Introduction

The first Biobank in Taiwan was officially established in 2005 in Academia Sinica (AS), the largest and most prestigious research institute of Taiwan ^10^. The purpose of this Biobank is to discover the hidden genetic diseases of Taiwanese people and promote their health. The project aimed to recruit two hundred thousand residents. So far, more than that number of people has taken part in the project by providing their personal information and details of their living habits. About half of the participants have also donated human biological materials that are deposited in the AS Biobank for study. Beginning from one Biobank, the number of Biobanks in Taiwan has increased to 31 in the last 13 years. Among these, three are population based and the rest are disease oriented.

The team at AS Biobank has published many scholarly papers ^11– 13^, mostly in the area of public health and statistical determinations of the nation’s health status. However, discoveries of genetic causes of disease and possible cures are still below expectation. The Ministry of Health and Welfare, under which these Biobanks are registered, started evaluation visits to all Biobanks two years ago to assess whether or not these Biobanks have functioned and produced results as originally expected.

The findings of these visits are that substantial financial monetary and personnel resources have been invested but the results are not as promising as hoped for, because: 1) all biobanks operate on their own without sharing information with others thus if any researcher needs certain tissue for research, they will have to check all biobanks till the data is found, 2) some biobanks have never received any requests for data 3) the cost of maintaining a biobank increases with accumulated tissues 4) some question the value of setting up too many biobanks for lack of research that ended up in wasting valuable resources. As a result, a new initiative has been introduced to integrate all Biobanks through data-sharing while each Biobank maintains its own uniqueness.

### Biobank Structural Innovation Project

The Ministry of Health and Welfare of Taiwan has initiated a structural innovation project to integrate all Biobanks in the areas of stored data. This project is called the Biobank Integrating Platform. The purpose is to promote data sharing and shorten the time of scientific and ethical review so that researchers can start their studies with a minimum of delay.

The first step is to create an intranet to gather detailed information on all Biobanks’ data and make it available to all other Biobanks. In this way, each Biobank no longer works on its own and is integrated in a coordinated structure and service. Each Biobank still functions as originally established but the bio-data is sent to the integrated platform for circulation to researchers. However, there is only one window or portal that researchers need to contact when seeking to access research data. Additionally, this platform ensures that donors’ personal identifiable information will not be available to any researcher in order to ensure their protection. However, doubt has been raised within the Ethics Governance Council (EGC) of the Taiwan Biobank about whether individuals’ privacy can be absolutely safeguarded.

Several challenges still need to be resolved. First, there is no bargaining forum for researchers who pay large fees for data. Each Biobank has a different scale of fees and some are expensive. Secondly, there is a lack of clarity about the intellectual property rights of the original institution versus the researcher and his/her institution in cases where novel research findings are discovered. Third, directors and or managers of Biobanks use large volumes of human biological materials from their own Biobanks, hence depriving the other researchers from accessing these limited resources Biobank. This has been criticized as a conflict of interest.

### Establishment of a scientific review committee

A scientific review committee has been set up to perform an initial review of all research protocols and then the Biobank’s Institutional Review Board (IRB) and EGC need only to do an expedited review to facilitate the review process. The bio-data fees are payable to the institutional Biobank that provides the data.

### Reflection

The establishment of the Biobank Integrating Platform facilitates researchers’ access to data, ensures the quality of human biological materials, saves resources and enhances the quality of research and promotes collaboration among researchers. This sample and data-sharing platform is new, and its effectiveness has already been demonstrated by an increase in applications for data. The integration is not to force all Biobanks into one; rather, each Biobank maintains its own strength, vision and uniqueness while opening up to all researchers through a common portal so that the goal of promoting health and curing diseases can be realized, and public health benefits can be maximized.

## Case study 2: Establishing the National Biorepository in Uganda: some regulatory and ethical uncertainties

### Introduction

In 2006, Uganda adopted a centralized model to scale-up its national HIV Early Infant Diagnosis (EID) programme. A HIV viral load monitoring (VL) programme was implemented in July 2014. Human biological materials such as dried blood spots (DBS) and plasma are collected from all health facilities in Uganda and delivered to HUBS. A HUB is a coordination center of the sub-district network serving approximately 20–40 health facilities where several referral tests are done, including: CD4+ counts, liver function test, renal function tests, complete blood counts etc. To date, there are 100 functional HUBS bringing together a network of over 2500 heath facilities. EID and VL human biological materials are transported from the HUBS to the Central Public Health Laboratory for testing ^14^. The total national coverage of both EID and VL for over 150,000 HIV exposed infants and 1,100,000 HIV patients on ART has resulted in the collection of over 1,000,000 remnant DBS and plasma human biological materials in a National Biorepository for future research. Approximately, 1,600 microbiological isolates are received from surveillance and epidemic investigations across various regions in Uganda. In September 2016, the National Biorepository proposed to set up a biorepository for appropriate storage of human biological materials in a retrievable manner for future research purposes and to foster both local and international research collaborations. The National Biorepository is owned by the Government of Uganda under the custodianship of Central Public Health Laboratories (CPHL). During 2017–2018, the National Biorepository has sought prior informed consent for long term storage and use of remnant clinical human biological materials, mainly from the centralized reference HIV early infant diagnosis (EID) and viral load programmes, as well as isolates of antimicrobial drug resistance surveillance and disease outbreak investigations. An informed consent statement has been added to the laboratory request forms. A Biorepository Governance Committee has been appointed to oversee the activities of the National Biorepository, provide direction on priority human biological materials and to store and regulate access to the repository resources. Plans are underway to create collaborations with universities and research institutions to promote human biological materials access. In addition, the National Biorepository will provide training in biorepository science to medical students and health workers.

### Planning and development

CPHL has set up a task force to develop a proposal to store remnant human biological materials ^15^. The proposal was submitted to an accredited Research Ethics Committee (REC) in Uganda. The protocol was reviewed and the feedback was that establishment of Biorepositories was outside the scope of ethics review by the REC. The REC advised that it would only be within its scope if a researcher intending to use the stored human biological materials applied for ethics review. Additionally, we were advised to submit the protocol to the Uganda National Council of Science and Technology (UNCST). The protocol was submitted to UNCST early in 2017, but no formal feedback was received until December 2018. Oversight of research involving humans as research participants in Uganda is done first at the organization level by RECs and second at national level by UNCST in collaboration with Uganda National Research Organization (UNHRO) ^16^. Unfortunately, UNCST currently has no regulations governing the establishment and operation of Biobanks/biorepositories. This has resulted in an unregulated proliferation of independent research Biobanks and/or biorepositories established to serve specific research interests in Uganda. Additionally, the regulatory body has apparently not yet mapped existing biorepositories/Biobanks in Uganda. As a consequence, the National Biorepository proposal and Standard Operating Procedures remain unapproved by UNCST.

### Informed consent

Implementation of informed consent in a setting with no regulations on Biobanking is challenging. National guidelines for research involving humans as research participants state that a specific informed consent form shall be used for human biological materials that are collected with the intention of being stored and used for future studies ^16^. This model offers the best protection for autonomy but has several limitations. It is difficult or impossible to gain specific consent, as future uses of the human biological materials and data are unknown at the time of diagnostic testing. Broad consent in cases where several possible future research uses are provided to research participants would be a good strategy to increase utilization of human biological materials and associated data and could foster international collaboration ^17^. Currently, UNCST is reviewing the Guidelines to offer guidance on the type of informed consent applicable to Biobanking institutions, especially for remnant human biological materials of clinical origin. Currently, the National Biorepository allows access of stored human biological materials to researchers who seek approval through an accredited REC to waive informed consent for the use of human biological materials for minimal risk research. This type of consent is however limited by the lack of national regulations. This also hinders collaborations.

### Community engagement

A stakeholder consultation meeting was conducted in 2018. The stakeholders comprised UNCST, civil society, lawyers from the Ministry of Justice and Constitutional Affairs, REC, district health officers, hospital directors, university lecturers and students and development partners. Information was shared about the National Biorepository such as Current Status and future prospects; its governance and legal and ethical issues. Resolutions from this meeting included: (a) Clinical and laboratory request forms should be modified to include a broad consent for storage and future use for research. (b) UNCST was tasked to write biorepository guidelines based on international standards. (c) For remnant human biological materials already in storage without consent, the National Biorepository should seek government advice through the attorney general. (d) UNCST was tasked to fast-track the compilation of Biobanking specific policies and guidelines.

### Reflection

National Regulatory Bodies are required to generate Biobanking Guidelines and Policies. Inadequate specialized ethics and regulatory knowledge seems to be the major cause of the lack of regulations or policies to guide Biobanking science in Uganda. Hence, education on Biobanking science and ethics in LMICs is required.

### Discussion

LMICs generally have weaker research capacity and governance mechanisms for Biobanks than high-income countries ^6, 18^. Human biological materials and data sharing from Biobanks are increasingly being used to support collaborative national and international health research. These approaches have the potential to increase scientific efficiency by maximizing the utility of human biological materials and data for researchers and funders ^8^. Managing data flows into and out of Biobanks gives rise to various ethical challenges which are exacerbated in some LMIC settings due to disparities in infrastructure, resources and capacity ^7^. National and or local regulatory authorities are required to develop Biobanking Guidelines ^17, 19^. In 2010, the Government of Western Australia through its Department of Health issued an operational directive that launched Guidelines for human Biobanks, genetic research databases and associated data that provides a set of principles and best practices to guide researchers and clinicians involved with Biobanks ^20^. Requesting appropriate informed consent in Biobanks has become a cornerstone for the collection of human biological materials and data for use in research, but this needs to be supported by relevant guidelines and legislation. For research involving Biobanking, government regulations and guidelines should identify key categories of information to be communicated to prospective participants during the consent process ^21, 22^. Various consent types ^12, 17^ have been recommended such as Consent waiver, Opt out, Opt in (Specific consent, Specific and broad consent), Broad consent and Dynamic consent. The establishment of Biobanks is an important step towards establishing national genomics research programmes. Maintaining these Biobanks and producing effective scientific outcomes based on Biobanking resources are not easy without a proper legislative, regulatory and governance framework. Good governance of a Biobank includes engaging with the public during the establishment of a Biobank and throughout the lifecycle of the Biobank ^23^.

## Conclusion

These two cases studies from different LMIC settings show that although in both settings there is strong awareness of the scientific and population health value of biorepositories, and strong commitment to their establishment, regulatory and ethical guidance show gaps that need to be addressed. Unfortunately, exact comparative categories of data could not be collected for each data bank and this should be remedied in future comparative studies. These gaps concern both the ethical acquisition of new human biological materials and the management of ethical access and use of such national resources in a way that is respectful of the donor communities, local regulations and legislation, and international best practices. Efforts need to be made nationally and internationally to create suitable enabling ethical governance of these valuable national and international resources.

## Data availability

### Underlying data

No data are associated with this article.

## References

[1] Abimiku A Mayne ES Joloba M: H3Africa Biorepository Program: Supporting Genomics Research on African Populations by Sharing High-Quality Biospecimens. *Biopreserv Biobank.* 2017;15(2):99–102. 10.1089/bio.2017.0005

[2] Lawlor RT Sluss PM Langer R: European, Middle Eastern, and African Society for Biopreservation and Biobanking (ESBB). 2012 Conference Session on Biobanking in Emerging Countries. *Biopreserv Biobank.* 2013;11(3):176–81. 10.1089/bio.2013.0017 24850095

[3] Sgaier SK Jha P Mony P: Public health. Biobanks in developing countries: needs and feasibility. *Science.* 2007;318(5853):1074–5. 10.1126/science.1149157 18006727

[4] Klingström T: Biobanking in emerging countries. *Biopreserv Biobank.* 2013;11(6):329–30. 10.1089/bio.2013.1161 24835361

[5] WMA - The World Medical Association: WMA Declaration of Taipei on Ethical Considerations regarding Health Databases and Biobanks [Internet].[cited 2019 May 20]. Reference Source

[6] Rudan I Marušić A Campbell H: Developing biobanks in developing countries. *J Glob Health.* 2011;1(1):2–4. 23198094 PMC3484738

[7] GFBR-2018-background-paper-FINAL.pdf [Internet]. [cited 2019 May 17]. Reference Source

[8] Bull S Cheah PY Denny S: Best Practices for Ethical Sharing of Individual-Level Health Research Data From Low- and Middle-Income Settings. *J Empir Res Hum Res Ethics.* 2015;10(3):302–13. 10.1177/1556264615594606 26297751 PMC4547207

[9] de Vries J Munung SN Matimba A: Regulation of genomic and biobanking research in Africa: a content analysis of ethics guidelines, policies and procedures from 22 African countries. *BMC Med Ethics.* 2017;18(1):8. 10.1186/s12910-016-0165-6 28153006 PMC5289015

[10] 衛生福利部. 衛生福利部單位網站 [Internet].衛生福利部. [cited 2019 Jun 8].2016 Reference Source

[11] Chen CH Yang JH Chiang CWK: Population structure of Han Chinese in the modern Taiwanese population based on 10,000 participants in the Taiwan Biobank project. *Hum Mol Genet.* 2016;25(24):5321–5331. 10.1093/hmg/ddw346 27798100 PMC6078601

[12] Ho CH: Rethinking Informed Consent in Biobanking and Biomedical Research: a Taiwanese Aboriginal Perspective and the Implementation of Group Consultation. *ABR.* 2017;9(4):353–365. 10.1007/s41649-017-0037-5

[13] Lin WY Chan CC Liu YL: Performing different kinds of physical exercise differentially attenuates the genetic effects on obesity measures: Evidence from 18,424 Taiwan Biobank participants. *PLoS Genet.* 2019;15(8):e1008277. 10.1371/journal.pgen.1008277 31369549 PMC6675047

[14] Kiyaga C Sendagire H Joseph E: Uganda's new national laboratory sample transport system: a successful model for improving access to diagnostic services for Early Infant HIV Diagnosis and other programs. *PLoS One.* 2013;8(11):e78609. 10.1371/journal.pone.0078609 24236026 PMC3827263

[15] Ssewanyana I Kiyaga C Nansumba H: Establishment of a National Health Laboratory Services Biorepository in Uganda. Central Public Health Laboratories.2016.

[16] UNCST: National Guidelines for Research involving Human as Research Participants. Uganda National Council for Science and Technology.2014 Reference Source

[17] Afifi N Betsou F Compton C: ISBER BEST PRACTICES INTERNATIONAL REVIEW BOARD.104. Reference Source

[18] Chen H Pang T: A call for global governance of biobanks. *Bull World Health Organ.* 2015;93(2):113–7. 10.2471/BLT.14.138420 25883404 PMC4339960

[19] Simeon-Dubach D Anisimov S Cohen Y: STANDARDS COMMITTEE REVIEWERS.36.

[20] OD299-Guidelines-for-human-biobanks-and-genetic-research-databases.pdf [Internet]. [cited 2019 May 17]. Reference Source

[21] McGuire AL Beskow LM: Informed consent in genomics and genetic research. *Annu Rev Genomics Hum Genet.* 2010;11:361–81. 10.1146/annurev-genom-082509-141711 20477535 PMC3216676

[22] Vaught J Lockhart NC: The evolution of biobanking best practices. *Clin Chim Acta.* 2012;413(19–20):1569–75. 10.1016/j.cca.2012.04.030 22579478 PMC3409343

[23] CE Revised Guidelines_Final_September 2017 (1).pdf [Internet]. [cited 2019 Ma y 17]. Reference Source

