# Supplementary material for: Role of a regulatory and governance framework in human biological materials and data sharing in National Biobanks: Case studies from Biobank Integrating Platform, Taiwan and the National Biorepository, Uganda
Source: Wellcome Open Res. 2020 Sep 1;4:171. Originally published 2019 Nov 12. [Version 2] doi: 10.12688/wellcomeopenres.15442.2 (PMC7479498; doi:10.12688/wellcomeopenres.15442.2)
Supplement: Peer review history

# Peer review: Reviewer response for version 2

**DOI:** 10.21956/wellcomeopenres.17885.r40222

**Published:** 2020-09-08

**Authors:** Jantina de Vries

**Affiliations:** 1 Department of Medicine, Faculty of Health Sciences, University of Cape Town, Cape Town, South Africa

Does the article adequately reference differing views and opinions?

Yes

Are all factual statements correct, and are statements and arguments made adequately supported by citations?

Yes

Is the Open Letter written in accessible language?

Partly

Where applicable, are recommendations and next steps explained clearly for others to follow?

Partly

Is the rationale for the Open Letter provided in sufficient detail?

Yes

Reviewer Expertise:

Bioethics, ethics of genomics, qualitative research methods

I confirm that I have read this submission and believe that I have an appropriate level of expertise to confirm that it is of an acceptable scientific standard.

# Peer review: Reviewer response for version 1

**DOI:** 10.21956/wellcomeopenres.16888.r37459

**Published:** 2020-01-29

**Authors:** Jantina de Vries

**Affiliations:** 1 Department of Medicine, Faculty of Health Sciences, University of Cape Town, Cape Town, South Africa

**Substantive issues**

*Taiwan case study*

 In the Taiwan case study you say that “substantial financial monetary and personnel resources have been invested but the results are not as promising as hoped for, *implying wastage of valuable resources.*” Do you mean to suggest that if there had been less wastage and more efficiency, the biobanks would have been more successful? Or do you mean to say that perhaps the entire idea of biobanking is a bit of a waste? It would be good if you could clarify this – not in the least because there are also other authors who have warned that the promises of biobanking may have been overplayed and that the expectations are unrealistic.

 For the Uganda case study, information is provided about the informed consent process, the regulatory approval process and community engagement. In order for these two case studies to be comparable for the reader, it would be good if they could both present similar types of information. Can the Taiwan case study perhaps be enriched with a description of those same items?

*Uganda case study*

 W.r.t. the Ugandan case study, the article describes that the biobank application was ‘not approved’. Wouldn’t it be more correct to say that it was ‘not reviewed’? From your explanation, it looks like the HREC determined that reviewing the establishment of a biobank fell outside of its mandate and so the suggestion is that they didn’t review it. (please note that your use of ‘we’ in that paragraph is different in style from the rest of the descriptions you give and you may want to change this). W.r.t. that case study – have you got any idea what is causing the delays in review by UNCST? It would be helpful for the article if you could spend a few more words describing the nature of your discussions with UNCST and what you attribute this delay to.

 In the Ugandan case study, there is some confusion about HIV DBS repository, the ‘proposal’ developed by CPHL the ‘national biorepository’ and what you refer to as ‘The Biorepository’. Please can you streamline this. Are these all one and the same repository or are these different ones? Do they exist already or are they still in development?

*Discussion*

 As it stands, the paper Discussion is more a summary of published literature than a discussion of the findings from both case studies. The Discussion should be enriched with a discussion of the observations and lessons learnt from the two case studies. For instance, I would have liked to learn more about how your understanding of the particular contexts enriches the international literature. What did you find or experience that is different from what others have published? Also, what is common and what is different in the two cases you present? Taiwan and Uganda are obviously two very different countries, on different continents with different languages, GDPs, cultures and scientific traditions. Are there any common lessons learnt?

**Issues of style and form**

 Throughout the manuscript, there is a shifting between different units of analysis that is not justified or explained. For instance, whilst the abstract concludes that “In general, LMICs have weaker research regulatory infrastructure and governance mechanisms for Biobanks than high-income Countries”, the work in the paper only focused on case studies in two countries (Uganda and Thailand) and one cannot use evidence from those two countries to make generalised statements of the regulations in all LMICs. The authors need to revise their manuscript to make sure that their observations and conclusions are pertinent to their observations in those two countries, and not extrapolate too broadly.

  Finally, there are many typographical and syntax errors in the document that need to be corrected by the authors. The authors particularly need to streamline their use of tenses – in some instances, both past and present tenses are used in the same paragraphs.

Does the article adequately reference differing views and opinions?

Yes

Are all factual statements correct, and are statements and arguments made adequately supported by citations?

Yes

Is the Open Letter written in accessible language?

Partly

Where applicable, are recommendations and next steps explained clearly for others to follow?

Partly

Is the rationale for the Open Letter provided in sufficient detail?

Yes

Reviewer Expertise:

Bioethics, ethics of genomics, qualitative research methods

I confirm that I have read this submission and believe that I have an appropriate level of expertise to confirm that it is of an acceptable scientific standard, however I have significant reservations, as outlined above.

# Response

**Published:** 2020-09-02

**Authors:** Hellen Nansumba

**Affiliations:** Central Public Health Laboratories, Uganda

**A Summary of responses to reviewers’ comments**

**1. Reviewers’ comment**

**In the Taiwan case study you say that “substantial financial monetary and personnel resources have been invested but the results are not as promising as hoped for, implying wastage of valuable resources.” Do you mean to suggest that if there had been less wastage and more efficiency, the bio-banks would have been more successful? Or do you mean to say that perhaps the entire idea of biobanking is a bit of a waste? It would be good if you could clarify this – not in the least because there are also other authors who have warned that the promises of biobanking may have been overplayed and that the expectations are unrealistic.**

Response to Reviewers’ comment

 Thank you for this comment. The section has been changed to read: The findings of these visits are that substantial financial monetary and personnel resources have been invested but the results are not as promising as hoped for, because:1) all bio-banks operate on their own without sharing information with others.  Thus, if any researcher needs certain tissue for research, she/he will have to check all bio-banks till the data is found, 2) some bio-banks have never received any requests for data 3) the cost of maintaining the bio-bank increases with accumulated tissues 4) some question the value of setting up too many bio-banks for lack of research that ended up wasting much of valuable resources.

**2. Reviewers’ comment**

**For the Uganda case study, information is provided about the informed consent process, the regulatory approval process and community engagement. In order for these two case studies to be comparable for the reader, it would be good if they could both present similar types of information. Can the Taiwan case study perhaps be enriched with a description of those same items?**

Response to Reviewers’ comment

 Thank you for this comment. This paper a summary of the presentation made at the 2018 GFBR and the Taiwan Case study did not highlight the informed consent process procedures and this information is unfortunately not available to us. We have acknowledged this as a limitation added a line to this effect, with a recommendation for future studies to use comparable data categories.

**3. Reviewers’ comment**

**W.r.t. the Ugandan case study, the article describes that the biobank application was ‘not approved’. Wouldn’t it be more correct to say that it was ‘not reviewed’? From your explanation, it looks like the HREC determined that reviewing the establishment of a biobank fell outside of its mandate and so the suggestion is that they didn’t review it. (please note that your use of ‘we’ in that paragraph is different in style from the rest of the descriptions you give and you may want to change this). W.r.t. that case study – have you got any idea what is causing the delays in review by UNCST? It would be helpful for the article if you could spend a few more words describing the nature of your discussions with UNCST and what you attribute this delay to.**

Response to Reviewers’ comment

 Thanks for this comment. This section has been revised to read: The protocol was reviewed and the feedback was that establishment of Biorepositories was outside the scope of ethics review by the REC. The REC advised that it would only be within its scope if a researcher intending to use the stored human biological materials applied for ethics review.

**4.** **Reviewers’ comment**

**In the Ugandan case study, there is some confusion about HIV DBS repository, the ‘proposal’ developed by CPHL the ‘national biorepository’ and what you refer to as ‘The Biorepository’. Please can you streamline this. Are these all one and the same repository or are these different ones? Do they exist already or are they still in development?**

Response to Reviewers’ comment

 Thank you. We have harmonized the word “Biorepository” in the document

**5. Reviewers’ comment**

**As it stands, the paper Discussion is more a summary of published literature than a discussion of the findings from both case studies. The Discussion should be enriched with a discussion of the observations and lessons learnt from the two case studies. For instance, I would have liked to learn more about how your understanding of the particular contexts enriches the international literature. What did you find or experience that is different from what others have published? Also, what is common and what is different in the two cases you present? Taiwan and Uganda are obviously two very different countries, on different continents with different languages, GDPs, cultures and scientific traditions. Are there any common lessons learnt?**

Response to Reviewers’ comment

 Thank you. The Discussion has been expanded to read:

 LMICs generally have weaker research capacity and governance mechanisms for Biobanks than high-income countries ^6^ ^, 18^. Human biological materials and data sharing from Biobanks are increasingly being used to support collaborative national and international health research. These approaches have the potential to increase scientific efficiency by maximizing the utility of human biological materials and data for researchers and funders ^8^ . Managing data flows into and out of Biobanks gives rise to various ethical challenges which are exacerbated in some LMIC settings due to disparities in infrastructure, resources and capacity ^7^ . National and or local regulatory authorities are required to develop Biobanking Guidelines ^17^ ^, 19^. In 2010, the Government of Western Australia through its Department of Health issued an operational directive that launched Guidelines for human Biobanks, genetic research databases and associated data that provides a set of principles and best practices to guide researchers and clinicians involved with Biobanks ^20^ . Requesting appropriate informed consent in Biobanks has become a cornerstone for the collection of human biological materials and data for use in research, but this needs to be supported by relevant guidelines and legislation. For research involving Biobanking, government regulations and guidelines should identify key categories of information to be communicated to prospective participants during the consent process ^21^ ^, 22^. Various consent types ^12^ ^, 17^have been recommended such as Consent waiver, Opt out, Opt in (Specific consent, Specific and broad consent), Broad consent and Dynamic consent. The establishment of Biobanks is an important step towards establishing national genomics research programmes. Maintaining these Biobanks and producing effective scientific outcomes based on Biobanking resources are not easy without a proper legislative, regulatory and governance framework. Good governance of a Biobank includes engaging with the public during the establishment of a Biobank and throughout the lifecycle of the Biobank ^23^

# Peer review: Reviewer response for version 1

**DOI:** 10.21956/wellcomeopenres.16888.r36980

**Published:** 2019-12-10

**Authors:** Nirmal Kumar Ganguly, Gautam Kumar Saha

**Affiliations:** 1 Policy Center for Biomedical Research, Translational Health Science and Technology Institute, Faridabad, India; 2 Apollo Hospitals Educational & Research Foundation, Hyderabad, Telangana, India

National Biobanks are essential for health system research. New biomedical technology is accelerating research in the discovery of new biomarkers which are subsequently used in predicting and the treatment protocols. Here biobanks play a pivotal role in identifying and validating such markers. The paper analyses how in the various countries that are emerging economies, like Taiwan and Uganda, are effectively using their biobank to further their health research. The paper is a good scientific read and points out the challenges like how patient data confidentiality have to be maintained as well sharing of the same have to be streamlined to gain maximum benefits. The article also mentions that this can be achieved through robust health research policy guidelines. The paper brings into light various aspects of biobanking and hence should be indexed.

Does the article adequately reference differing views and opinions?

Yes

Are all factual statements correct, and are statements and arguments made adequately supported by citations?

Yes

Is the Open Letter written in accessible language?

Yes

Where applicable, are recommendations and next steps explained clearly for others to follow?

Yes

Is the rationale for the Open Letter provided in sufficient detail?

Yes

Reviewer Expertise:

Public Health, Microbiology, Biotechnology, Cardiovascular and Infectious Diseases

We confirm that we have read this submission and believe that we have an appropriate level of expertise to confirm that it is of an acceptable scientific standard.
